# Idiopathic Granulomatous Mastitis or Breast Cancer? A Comparative MRI Study in Patients Presenting with Non-Mass Enhancement

**DOI:** 10.3390/diagnostics13081475

**Published:** 2023-04-19

**Authors:** Fatma Nur Soylu Boy, Gul Esen Icten, Yasemin Kayadibi, Iksan Tasdelen, Dolunay Alver

**Affiliations:** 1 Department of Radiology, Fatih Sultan Mehmet Training and Research Hospital, 34758 Istanbul, Turkey; 2 Senology Research Institute, Acibadem Mehmet Ali Aydınlar University, 34457 Istanbul, Turkey; 3 Department of Radiology, School of Medicine, Acibadem Mehmet Ali Aydınlar University, 34457 Istanbul, Turkey; 4 Department of Radiology, Cerrahpasa Medical Faculty, Istanbul University-Cerrahpasa, 34320 Istanbul, Turkey; 5 Department of General Surgery, Fatih Sultan Mehmet Training and Research Hospital, 34758 Istanbul, Turkey

**Keywords:** granulomatous mastitis, breast cancer, MRI, non-mass enhancement

## Abstract

Objective: To compare and determine discriminative magnetic resonance imaging (MRI) findings of idiopathic granulomatous mastitis (IGM) and breast cancer (BC) that present as non-mass enhancement. Materials and Methods: This retrospective study includes 68 IGM and 75 BC cases that presented with non-mass enhancement on breast MRI. All patients with a previous history of breast surgery, radiotherapy, or chemotherapy due to BC or a previous history of mastitis were excluded. On MRI images, presence of architectural distortion skin thickening, edema, hyperintense ducts containing protein, dilated fat-containing ducts and axillary adenopathies were noted. Cysts with enhancing walls, lesion size, lesion location, fistulas, distribution, internal enhancement pattern and kinetic features of non-mass enhancement were recorded. Apparent diffusion coefficient (ADC) values were calculated. Pearson chi-square test, Fisher’s exact test, independent t test and Mann–Whitney U test were used as needed for statistical analysis and comparison. Multivariate logistic regression model was used to determine the independent predictors. Results: IGM patients were significantly younger than BC patients (*p* < 0.001). Cysts with thin (*p* < 0.05) or thick walls (*p* = 0.001), multiple cystic lesions, (*p* < 0.001), cystic lesions draining to the skin (*p* < 0.001), and skin fistulas (*p* < 0.05) were detected more often in IGM. Central (*p* < 0.05) and periareolar (*p* < 0.001) location and focal skin thickening (*p* < 0.05) were significantly more common in IGM. Architectural distortion (*p* = 0.001) and diffuse skin thickening (*p* < 0.05) were associated with BC. Multiple regional distribution was more common in IGM, whereas diffuse distribution and clumped enhancement were more common in BC (*p* < 0.05). In kinetic analysis, persistent enhancement was more common in IGM, whereas plateau and wash-out types were more common in BC (*p* < 0.001). Independent predictors for BC were age, diffuse skin thickening and kinetic curve types. There was no significant difference in the diffusion characteristics. Based on these findings, MRI had a sensitivity, specificity and accuracy of 88%, 67.65%, and 78.32%, respectively, in differentiating IGM from BC. Conclusions: In conclusion, for non-mass enhancement, MRI can rule out malignancy with a considerably high sensitivity; however, specificity is still low, as many IGM patients have overlapping findings. Final diagnosis should be complemented with histopathology whenever necessary.

## 1. Introduction

Idiopathic granulomatous mastitis (IGM) is a rare inflammatory disease of the breast. Although IGM is a benign process, its complex clinical and heterogeneous radiologic presentation, which sometimes resembles breast cancer, may lead to difficulty in diagnosis [1,2,3,4,5]. Mammography and/or ultrasonography (US) are the usual diagnostic examinations. Especially considering the young and dense breast structure of the cases presenting with the suspicion of IGM, mammography generally provides non-specific findings (skin thickening, ill-defined asymmetrical densities, architectural distortion) that is often not indistinguishable from inflammatory breast cancer, except for the detection of microcalcification [3,6,7]. On the other hand, US is often the first-choice imaging method in these patients, demonstrating more specific findings than mammography, with finger-like collection areas that tend to merge. The sensitivity, specificity, and accuracy of B-mode ultrasonography in the differential diagnosis of IGM or malignancy were 82.8%, 77.1%, and 81.2%, respectively [3,8]. Although sonographic findings are often sufficient, more may be needed to detect extension of the affected area and additional findings due to an overlap with malignancy. In such cases, magnetic resonance imaging (MRI) is used as an advanced imaging tool that can suggest the nature of the illness and demonstrate the extent of the underlying disease [9,10,11].

The most common MRI findings of IGM are non-mass enhancement and cystic lesions that show rim enhancement in dynamic contrast-enhanced (DCE) images [11,12,13]. However, the interpretation of MRI can still be challenging for the differentiation of IGM and breast cancer, especially in cases with non-mass enhancement. The number of studies that compare MRI findings of IGM and breast cancer is limited in the literature [10,12,13,14]. Although diffusion-weighted imaging has also been evaluated in the differentiation of malignancy, it has been reported that diffusion restriction can also be detected due to necrotic material and microabscesses in the IGM [15,16,17]. This study was designed to compare the MRI findings of IGM and breast cancer that present as non-mass enhancement and to determine the discriminative features that could help in differential diagnosis.

## 2. Materials and Methods

### 2.1. Study Design 

This multicenter retrospective study was conducted in the radiology clinics of two research hospitals and was approved by the institutional review boards with a waiver of patient informed consent. Patient files were retrospectively evaluated to list all the patients that received a pathological diagnosis of IGM or breast cancer between January 2020 and April 2022. From this list, patients who had an MRI evaluation before the onset of treatment, the images of which could be accessed from the local PACS, were determined. MRI reports of these patients were evaluated. All IGM patients with non-mass enhancement with or without cystic lesions and all invasive breast cancer patients whose disease presented predominantly as non-mass enhancement were included in the study. Patients with mass enhancement were excluded because they usually do not create a diagnostic challenge, and we planned to concentrate on patients with non-mass enhancement specifically. Furthermore, all patients with a previous history of breast surgery, radiotherapy, or chemotherapy due to breast cancer or a previous history of mastitis were excluded. Patients with incomplete or technically inadequate MRI examinations were also excluded from the study. An equal number of patients were aimed to be included in each group, but due to the exclusion of some cases, the final study group comprised 143 patients (68 with IGM and 75 with breast cancer). In all cases, histopathologic diagnoses were obtained by US-guided core needle biopsy, which were performed after the MRI examination.

### 2.2. MRI

MRI was performed using a 1.5 T system of the same vendor (Optima MR450w, GE Healthcare) and dedicated 8-channel breast coil in prone position at both centers. Multiparametric MR images of bilateral breasts were obtained in the axial plane, including the following sequences: T1-weighted fast spin-echo, T2-weighted fat-suppressed fast spin-echo, diffusion-weighted echo-planar images (DWI) with diffusion gradients at b values of 0 and 800, and T1-weighted turbo 3D gradient echo dynamic contrast-enhanced images (one before and six after administration of IV contrast material). The contrast agent gadoterate meglumine (Dotarem, Guerbet) was automatically injected at a rate of 2.0 mL/s. The contrast dose (0.1 mmol/kg) was based on the patient’s weight.

### 2.3. Image Interpretation

Anonymized MR images were evaluated by two experienced breast radiologists (with 25 years and 15 years of experience). Radiologists interpreted the images in consensus.

Evaluation was based on the 5th edition of Breast Imaging Reporting and Data System (BI-RADS^®^) MRI lexicon of the American College of Radiology [18]. Some additional features such as dilated fat containing ducts or fistulas were also examined as detailed below: 

In the non-enhanced series, the amounts of fibroglandular tissue, hyperintense ducts containing proteinous secretion on T1-W images, architectural distortion, skin thickening, and dilated fat containing ducts were noted. Skin thickening (>3 mm) was grouped as regional, diffuse, or periareolar. Fat-containing ducts were defined as ducts that had the same signal intensity with fat in all sequences, including fat suppression. Edema was classified as perilesional, subcutaneous, diffuse, prepectoral, and presternal on T2-weighted images. Presence and number of cysts were recorded. 

In the contrast-enhanced series, lesion location (upper outer, upper inner, lower outer, lower inner quadrants, central, subareolar and periareolar) was recorded. Maximum diameter as well as the distribution (focal, linear, segmental, regional, multiple regional, diffuse) and internal enhancement pattern (homogeneous, heterogeneous, clumped and clustered ring) of the enhancing lesions were noted. Kinetic curve analysis was performed by placing a standardized circular ROI at the enhancing areas. The early phase (slow, medium and rapid) and late-phase kinetics (persistent, plateau, wash-out) were recorded. In the presence of cystic lesions with enhancing walls, they were categorized according to the irregularity and the thickness of their walls (thin: ≤0.5 mm, thick: >0.5 mm). Additionally presence of fistulas, cystic lesions that were draining directly to the skin, skin retraction or invasion, and nipple retraction or invasion were recorded. Suspicious axillary lymph nodes were noted. 

DWI and apparent diffusion coefficient (ADC) maps were analyzed, and a visual scale between 0 and 3 (0: none, 1: mild, 2: moderate and 3: marked restriction) was used to determine the degree of diffusion restriction. Mean ADC values of 3 measurements were obtained by placing standardized circular region of interest (ROI) on ADC maps, corresponding to areas of non-mass enhancement on DCE images.

### 2.4. Statistical Analysis

The Shapiro–Wilk test was used to test the normality of the data. Descriptive analyses were presented using mean ± SD for normally distributed data and median (min-max) for non-normally distributed data or *n* (%) for categorical variables. Categorical data were compared with Pearson chi-square test and Fisher’s exact test. We used the chi-square test if ≤20% of expected cell counts were less than 5, and we used Fisher’s exact test if >20% of expected cell counts were less than comparisons of continuous data between GM and breast cancer, which were performed by independent t test for normally distributed data and Mann–Whitney U test for non-normally distributed data. Variables with statistically significant differences in univariate analysis were included in multivariate model to determine the independent predictors for breast cancer. Odds ratio (OR) with corresponding 95% confidence intervals (95% CIs) was reported. Statistical analysis was performed with IBM SPSS Statistics for Windows, Version 23.0 (IBM Corp., Armonk, NY, USA). Two-sided *p* value less than 0.05 was considered statistically significant.

## 3. Results 

The patients’ ages ranged between 23 and 58 in the IGM group (mean ± SD: 37.1 ± 8.2) and between 27 and 80 in the breast cancer group (mean ± SD: 47.6 ± 10.5); the difference was statistically significant (*p* < 0.001). Subtypes of breast cancer were as follows: ductal carcinoma in situ (*n* = 10), invasive ductal carcinoma (*n* = 47), invasive lobular carcinoma (*n* = 14), invasive ductal and lobular carcinoma (*n* = 3), and metaplastic carcinoma (*n* = 1).

### 3.1. Non-Enhanced Images

**Figure 1:**
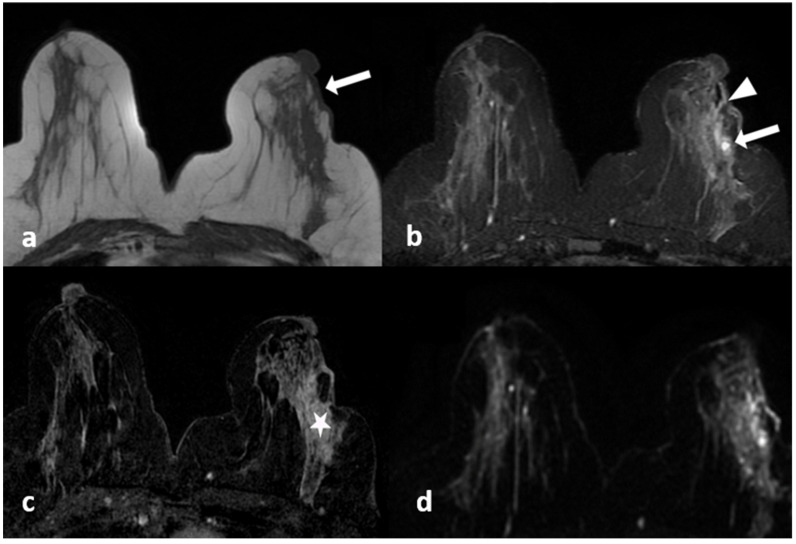
A 39-year-old woman with histopathologically proven idiopathic granulomatous mastitis involving the left breast. (**a**) T1-weighted fast spin echo axial MR image of the patient reveals a parenchymal asymmetry in the upper outer quadrant of the left breast and skin retraction with focal skin thickening (arrow); (**b**) axial fat-saturated T2-weighted fast spin echo MR image, corresponding to (**a**) a small cystic lesion (arrow) within an area of signal increase in parenchyma associated with edema; a fistulae formation is seen as hyperintense linear area extending to the skin (arrowhead). (**c**) Axial contrast-enhanced subtracted image visualizes a heterogeneous enhancement in the parenchyma (star), at the same level with (**a**,**b**,**d**): axial diffusion-weighted image shows hyperintense restricted area corresponding to (**a**–**c**).

**Figure 2:**
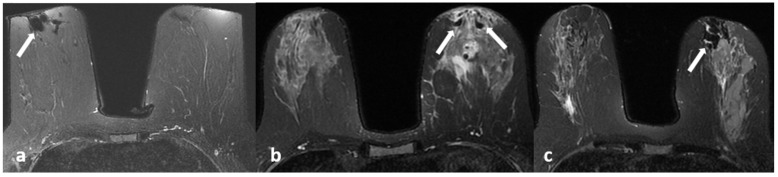
(**a**–**c**) Fat containing dilated ducts (arrows) on fat-suppressed T2-weighted images in three different patients.

**Figure 3:**
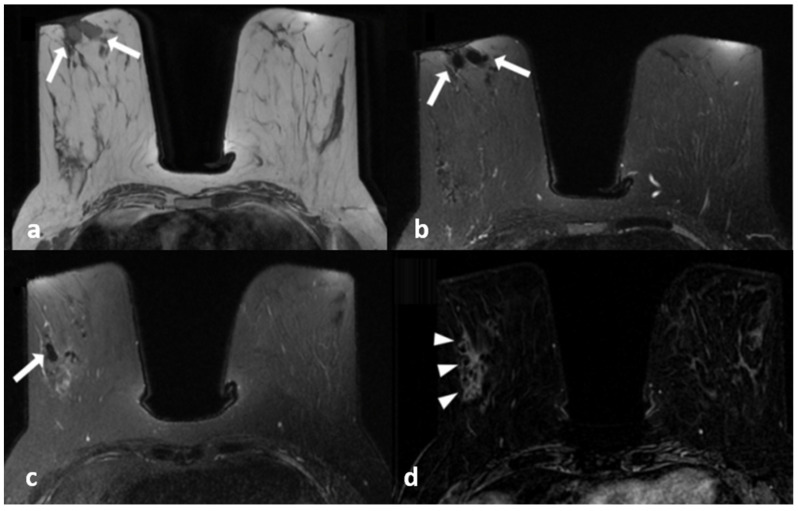
A 45-year-old woman with histopathologically proven idiopathic granulomatous mastitis involving the right breast. (**a**) Precontrast T1-weighted fast spin echo axial MR image of the patient shows dilated ducts with a high signal intensity in retroareolar region (arrows). (**b**) Axial fat-saturated T2-weighted image corresponding to (**a**), which reveals that the dilated duct has a signal loss in accordance with the fat contained (arrows). (**c**) Contrast-enhanced subtracted image demonstrates a non-mass enhancement with a clustered ring pattern in the upper outer quadrant of the right breast (arrowhead). (**d**) T2-weighted fat-saturated image corresponding to (**c**) also demonstrates the dilated ducts containing fat intensity within the enhanced area (arrow).

Architectural distortion was much more common in breast cancer (34.7%) compared to IGM (10.3%), (*p* = 0.001). IGM presented with focal skin thickening more often compared to breast cancer (*p* < 0.05), (Figure 1), whereas diffuse skin thickening was more common in breast cancer (*p* < 0.05). Dilated fat-containing ducts were more frequent in IGM (16.2%) compared to breast cancer (5.3%), but the difference was not statistically significant (Table 1 and Figure 2 and Figure 3). There was no significant difference between two groups in terms of amount of fibroglandular tissue, presence or absence of edema or the location of edema. Diffusion restriction based on both visual scale findings and ADC values showed no statistically significant difference (Table 2). 

### 3.2. Contrast-Enhanced Images

Mean lesion size was calculated as 72 mm (with a range of 11–154 mm) for IGM cases and 59 mm (with a range of 16–128 mm) for breast cancer patients (*p* > 0.05). 

**Figure 4:**
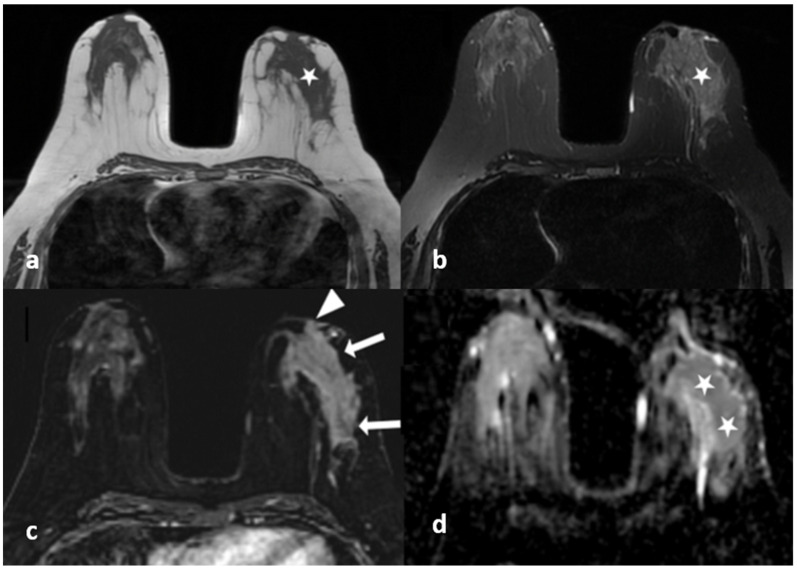
A 40-year-old woman with histopathologically proven idiopathic granulomatous mastitis involving the left breast. (**a**) T1-weighted fast spin echo axial MR image of the patient shows mild parenchymal asymmetry in the upper outer quadrant of the left breast (star). (**b**) Axial fat-saturated T2-wieghted fast spin echo MR image, corresponding to (**a**), which reveals a moderate signal increase in the parencyma (star). (**c**) Axial contrast-enhanced subtracted image demonstrates a heterogeneous enhancement in the parenchyma (arrows) and a pathologic enhancement extending to the skin in the periareolar region (arrowhead). (**d**) ADC map at the same location shows a moderate signal decrease (stars) revealing restriction of diffusion in the area corresponding to (**a**–**c**).

Central location was mostly seen in IGM with a significant difference (*p* < 0.05) and an OR of 0.14 (0.02–0.74). Periareolar location was also significantly more common in IGM (*p* < 0.001) (Figure 4).

**Figure 5:**
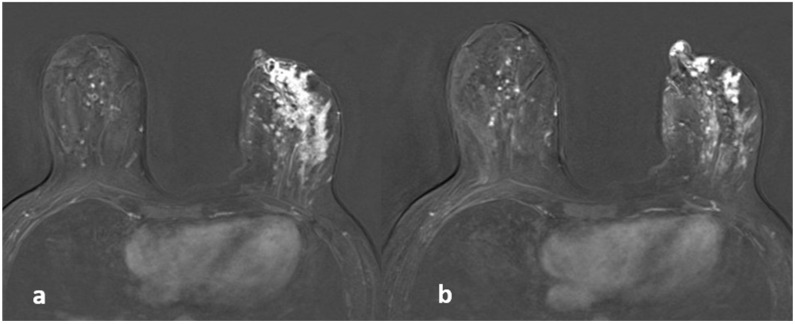
A 42-year-old woman with histopathologically proven invasive ductal carcinoma involving the left breast. (**a**,**b**) Contrast-enhanced subtracted images show segmental enhancement with clumped internal enhancement pattern extending into the nipple in the left breast.

There was a significant difference regarding the distribution of non-mass enhancement in the multiple regional and diffuse distribution categories (Table 3). Multiple regional distribution was more common in IGM, whereas diffuse distribution was more common in breast cancer (*p*: 0.048). There was no difference in terms of linear or segmental distribution patterns. In terms of internal enhancement patterns, there was a significant difference only in the clumped pattern (Figure 5), which was more common in breast cancer (22.7% versus 4.4%) (*p* < 0.05). Clustered ring pattern was seen in 40 patients with IGM (58.8%) and 38 patients with breast cancer (50.7%); the difference was not statistically significant (Figure 5).

Kinetic curve analysis showed significant difference only in the late-phase findings (Table 4). Persistent enhancement was more common in IGM, while plateau and wash-out patterns were more common in breast cancer (*p* < 0.001). 

**Figure 6:**
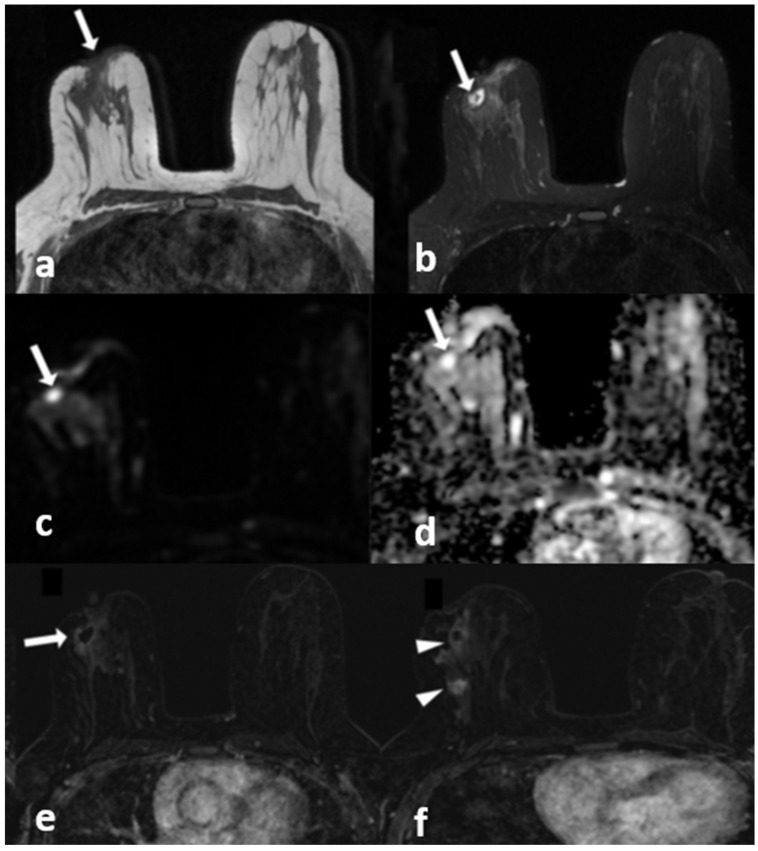
A 42-year-old woman with histopathologically proven invasive ductal carcinoma involving the right breast. (**a**) T1-weighted fast spin echo axial MR image of the patient shows parenchymal asymmetry in the retroareolar region along with nipple retraction (arrow). (**b**) Axial fat-saturated T2-weighted fast spin echo MR image, corresponding to (**a**), which reveals a cystic lesion (arrow) with a moderate signal increase in the parenchyma. (**c**) Diffusion-weighted and (**d**) ADC map show that the cystic lesion had no restriction of diffusion (arrows). (**e**) Contrast-enhanced subtracted image demonstrates a slight thin wall enhancement around the cystic lesion (arrow); (**f**) following the inferior image to (**e**) shows similar contiguous lesions demonstrating peripheral slight enhancement in the adjacent parenchyma (arrowheads).

Cystic lesions with enhancing walls were more diagnostic for IGM in general (Table 5). Cysts with thin (*p* < 0.05) or thick walls (*p* = 0.001) as well as multiple cysts with enhancing walls (*p* < 0.001) were significantly more common in IGM (Figure 6). Multiple cystic lesions, cystic lesions draining to the skin and skin fistulas were detected more often in IGM with a significant difference (*p* < 0.001, *p* < 0.001 and *p* < 0.05 respectively). The number of cases with cysts with irregular walls did not show any difference.

The number of cases with retraction or invasion in the skin or the nipple as well as those with suspicious axillary lymphadenopathies showed no difference between two diseases.

### 3.3. Overall Evaluation

Independent predictors for breast cancer were the age of the patient, diffuse skin thickening and kinetic curve types of plateau and wash-out in backward stepwise multivariate model (Table 6). Cystic lesions, multiple cystic lesions and central location were negatively associated with breast cancer.

Based on these results, we evaluated the findings of all patients again blinded to the histopathological results and tried to give a radiological diagnosis of IGM or breast cancer. Findings were re-evaluated according to the backward stepwise model; age over 50, diffuse skin thickness, and plateau or wash-out in the kinetic analysis were classified as breast cancer; age less than 50 years, containing cystic lesions and central localization were classified as IGM. We achieved a sensitivity of 88%, specificity of 67.65%, and accuracy of 78.32% for the detection of breast cancer. Positive (PPV) and negative (NPV) predictive values were 75% and 83.64%, respectively. If architectural distortion, non-mass enhancement with diffuse distribution or clumped internal enhancement pattern were also included in the malignancy criteria, sensitivity, specificity, accuracy, PPV and NPV were 97.33%, 55.88%, 79.02%, 70.87%, 95%, respectively (Table 7). The most common cause of false positive results was plateau type of kinetics (16/68 patients). However, it was also present in 36/75 breast cancer patients, and it was the only malignant finding in 10 cases.

## 4. Discussion 

In this study, we compared the MRI findings of IGM and breast cancer patients presenting with non-mass enhancement on breast MRI and tried to determine if there were any findings that could help us in the differential diagnosis. We found that young age, single or multiple cysts with enhancing walls, cysts that drained to the skin, skin fistulas, focal skin thickening, central or periareolar location, and non-mass enhancement with multiple regional distribution were features that were significantly more common in IGM. On the other hand, breast cancer patients were more likely to be older and had significantly more architectural distortion, diffuse skin thickening, non-mass enhancement with diffuse distribution and clumped internal enhancement. The persistent type of kinetic curve was more common in IGM, while plateau and washout types of kinetics were typical for breast cancer. Studies that compare the MRI findings of IGM and breast cancer are limited in the literature [10,12,13,14]. To the best of our knowledge, this is the first study involving such a large number of patients in this regard. 

We included only cases with non-mass enhancement in this study. Breast cancers presenting as mass lesions on MRI are usually more typical and are easier to differentiate from IGM. However, it has been reported that 57% of nonpalpable breast cancers demonstrate non-mass enhancement on MRI [19]. IGM also usually presents as non-mass enhancement that shows segmental or regional distribution on MRI [10,15,17]. In our daily clinical practice, we noticed that the cases with non-mass enhancement were much more difficult to differentiate, especially if they did not have coexisting cystic lesions. This was the reason we conducted this study. 

IGM is characterized by abscess formations, and it is not surprising that significantly more cystic lesions with enhancing walls were observed in IGM patients. This finding is similar to previous reports [10,12,13,20,21]. Fistula formation and sinus tracts are also common findings in IGM [20,21]. In our IGM group, 30.9% of patients had lesions that were directly draining into the skin, and the rate of fistula formation was 20.6%. However, three lesions (5.3%) draining directly into the skin and four lesions (6.7%) with fistula formation were detected in breast cancer patients.

In this study, focal skin thickening was more common in IGM (*p* < 0.05), while diffuse skin thickening was more common in breast cancer with an OR of (95% CI) 26,1 (2.1–314.7). Diffuse thickening was mostly detected in inflammatory breast cancer patients. In an MRI study by Renz et al. that compared breast cancer and acute mastitis, diffuse skin thickening was more common in inflammatory breast cancer, with no statistical significance [22]. A focal inflammatory response can be seen in the skin adjacent to IGM lesions. In our opinion, IGM should be more widespread and severe in order to affect the skin diffusely, whereas breast cancer may lead to diffuse skin thickening readily by lymphatic blockage [23,24]. 

Protein secretions in the ducts characterized by T1 hyperintensity were reported to be a possible etiologic factor for IGM [25]. This finding was rare in both groups in our study and showed no statistical difference. However, we noticed that some patients had fat containing dilated ducts, characterized by fat intensity in all sequences. This finding has not been discussed previously in the literature. Fat-containing ducts were much more common in IGM (16.2%) compared to breast cancer (5.3%), although the difference was not significant (*p* = 0.054). Since IGM is seen mostly at postpartum period, fat-containing dilated ducts may be due to the altered biochemical content of the lactiferous ducts. It may just be a finding associated with recent lactation, or the leakage of this secretion may be an underlying etiologic factor. We think that this finding should be evaluated further in future studies with larger numbers of patients.

Altunkeser and Gautier reported retroareolar location in 12% and 27% of IGM cases in their studies, respectively [20,26]. In this study, pure retroareoalar involvement was detected in only three IGM cases and one breast cancer case. However, periareolar location was significantly more common in IGM (60.3% and 18.7% for IGM and breast cancer, respectively) (*p* < 0.001), and the central part of the breast was also a frequent site of involvement for IGM with an OR of 0.069 (0.01–0.469). We have not encountered an analysis of central or periareolar location of IGM lesions in previous studies.

Segmental distribution has a high PPV for breast cancer; however, it is also one of the most common patterns of distribution for non-mass enhancement in IGM [10,14,15,17,27,28,29]. Consistent with the literature, it was the most common pattern in our study for both groups with no significant difference. Multiple regional distribution was the second most common pattern in IGM, and the difference was significant (*p* < 0.05). On the other hand, diffuse distribution was significantly more common in breast cancer (*p* < 0.05). Altunkeser et al. reported similar results, although there was no significant difference in their study, which included only 58 patients [26]. Our study revealed that, in addition to diffuse distribution pattern, architectural distortion, and clumped internal enhancement were also findings associated with breast cancer. 

Clustered ring enhancement is another finding that is closely associated with malignancy. It has been reported in recent studies that this finding was seen in up to 63% of the BC cases [29,30,31]. Clustered ring enhancement was the most common internal enhancement pattern in our study (58.8% in IGM and 50.7% in breast cancer), followed by heterogeneous enhancement (35.3% versus 26.7%, respectively). Neither of them were statistically significant. Clumped pattern was significantly more common in breast cancer (4.4% versus 22.7% respectively). This confirms the results of other studies in the literature [10,28,29,30,31,32]. 

There are conflicting results for DWI in IGM. Even though some authors have stated that IGM lesions show restricted diffusion [15,33], a recent report declared that DWI and ADC measurements are not helpful for differentiating IGM from breast cancer [17]. Our study is consistent with the latter report, as neither visual evaluation nor ADC measurements showed any statistical difference between the two diseases. 

As IGM is known for its likelihood to mimic breast cancer, correct evaluation of MRI features of this disease is crucial. Among the series evaluating comparatively between IGM and malignancy in the literature, the study closest to our series numerically is the study of Qu et al. [12]. This study compared MRI findings of non-calcified ductal carcinoma in situ (*n* = 36) and granulomatous mastitis (*n* = 33) cases. In this study, non-mass enhancement, clustered ring enhancement, ring size, rapid contrast enhancement in univariate analysis, internal enhancement, and rapid staining in multivariate analysis were found to have significant differences (AUC = 0.867, (95% CI, 0.748–0.943)). Considering ring size (cut of 7 mm), the sensitivity of MR was 81.8%, and the specificity was 82.1% [12]. The most significant difference in our study is that we worked with 143 cases, and only lesions showing non-mass staining were included. Our findings showed that 97.33% of breast cancer cases were either older than 50 years of age or demonstrated at least one finding that is suspicious for malignancy (diffuse skin thickening, architectural distortion, diffuse distribution, clumped enhancement, plateau or washout kinetics). Therefore, IGM should be a diagnosis of exclusion. In the absence of malignant findings, the presence of cysts with enhancing walls, skin fistula, central or periareolar location, and non-mass enhancement with multiple regional distribution may suggest IGM in a young patient (<50 years).

This study has some limitations. First, it is a retrospective study. Second, the study was performed blinded to clinical as well as other radiological findings. Future prospective studies investigating the role of MRI in routine clinical practice may be worthwhile. A strong point for this study was the large number of patients. IGM is a rare disease in many parts of the world, and this is the largest study in the literature on differential diagnoses of IGM and breast cancer on MRI. However, larger studies are still needed in order to establish more definitive criteria and to investigate other findings such as dilated fat-containing ducts.

In conclusion, according to our results, we can suggest that IGM is likely to present with one or multiple cystic lesions with enhancing walls, showing central or periareolar location and non-mass enhancement with multiple regional distribution and a persistent type of kinetics. Old age, architectural distortion, diffuse skin thickening, non-mass enhancement with diffuse distribution or clumped internal enhancement pattern with plateau, and wash-out kinetics should be warning signs for malignancy. The absence of these suspicious criteria can rule out malignancy with a considerably high NPV; however, PPV is still low, as many IGM patients have overlapping findings. A final diagnosis should be complemented with histopathology whenever necessary.

## Figures and Tables

**Table 1:** Non-enhanced findings of MRI for IGM and breast cancer (BC).

Nonenhanced Findings	IGM *n* (%)	BC *n* (%)	*p* Value
Amount of fibroglandular tissue			0.936
Tip A	7 (10.3)	7 (9.3)	
Tip B	28 (41.2)	28 (37.3)	
Tip C	29 (42.6)	36 (48.1)	
Tip D	4 (5.9)	4 (5.3)	
Architectural distortion	7 (10.3)	26 (34.7)	**0.001**
Hyperintense ducts	4 (5.9)	2 (2.7)	0.424
Fat containing ducts	11 (16.2)	4 (5.3)	0.054
Oedema	54 (79.4)	54 (72)	0.303
Perilesional	39 (57.4)	49 (65.3)	0.327
Subcutaneous	8 (11.8)	11 (14.7)	0.610
Diffuse	14 (20.6)	18 (24)	0.625
Prepectoral	27(39.7)	20 (26.7)	0.097
Presternal	0 (0)	1 (1.3)	0.999
Skin thickening	50 (73.5)	41 (54.6)	
Focal	32 (47.1)	20 (26.7)	**0.011**
Diffuse	3 (4.4)	11 (14.7)	**0.049**
Periareolar	27 (39.7)	21 (28)	0.139

Numbers in parentheses represent percentages.

**Table 2:** DWI findings.

ADC (10^−3^ mm^2^/s)	IGM	BC	*p*
ADC, median (min–max)	1038 (781–1557)	1023.5 (704–1523)	0.397
Standard deviation, median (min–max)	78.5 (21–740)	93 (10–397)	0.110

Numbers in parentheses in the visual diffusion restriction section represent percentages.

**Table 3:** Lesion distribution and internal enhancement characteristics of lesions presenting as non-mass enhancement.

**Distribution**	**IGM** ***n* (%)**	**BC** ***n* (%)**	***p* Value**
Focal	2 (2.9) ^a^	4 (5.3) ^a^	**0.048**
Linear	1 (1.5) ^a^	4 (5.3) ^a^	
Segmental	30 (44.1) ^a^	36 (48) ^a^	
Regional	9 (13.2) ^a^	6 (8) ^a^	
Multi-regional	22 (32.4) ^a^	12 (16) ^b^	
Diffuse	4 (5.9) ^a^	13 (17.3) ^b^	
**Internal Enhancement**	**IGM**	**BC**	***p* Value**
Homogeneous	1 (1.5) ^a^	0 (0) ^a^	**0.006**
Heterogeneous	24 (35.3) ^a^	20 (26.7) ^a^	
Clumped	3 (4.4) ^a^	17 (22.7) ^b^	
Clustered ring	40 (58.8) ^a^	38 (50.7) ^a^	

Numbers in parentheses represent percentages. Same letters (a, a) in a row denote the lack of statistically significant difference, different letters (a, b) denote statistically significant difference in binary analysis.

**Table 4:** Kinetic features in DCE MRI for differentiating IGM from breast cancer.

DCE Parameters	IGM	BC	*p*
Early phase			
Slow	18 (26.4) ^a^	20 (26.6) ^a^	0.352
Medium	24 (35.2) ^a^	34 (45.3) ^a^	
Rapid	26 (38.2) ^a^	21 (28.0) ^a^	
Late phase			
Persistent	40 (58.8) ^a^	19 (25.3) ^b^	**<0.001**
Plateau	20 (29.4) ^a^	38 (50.6) ^b^	
Washout	8 (11.7) ^a^	18 (24.0) ^b^	

Numbers in parentheses represent percentages. Same letters in a row denote the lack of statistically significant differences.

**Table 5:** Cystic lesion characteristics for IGM and breast cancer (BC).

Cystic Lesion Characteristics	IGM	BC	*p*
Cystic lesion	50 (73.5)	19 (25.3)	**<0.001**
*Thin walls*	17 (25)	6 (8)	**0.012**
*Thick walls*	24 (35.3)	8 (10.7)	**0.001**
*Irregular walls*	9 (13.2)	9 (12)	0.805
*Multiple cystic lesions*	41 (60.3)	8 (10.7)	**<0.001**
*Cyst draining to the skin* *Fistula*	21 (30.9) 14 (20.6)	3 (4) 5 (6.7)	**<0.001** **0.014**

**Table 6:** A backward stepwise multivariate logistic regression analysis to determine associated factors with BC.

Variables	OR (95% CI)	*p*
Age	1.150 (1.073–1.233)	**<0.001**
Cystic lesion	0.238 (0.060–0.943)	**0.041**
Multiple cystic lesion	0.088 (0.017–0.448)	**0.003**
Diffuse skin thickening	10.294 (1.342–78.981)	**0.025**
Central location	0.147 (0.029–0.744)	**0.021**
DCE Kinetic curves		
Persistent	Reference	-
Plateau	14.497 (3.698–56.838)	**<0.001**
Washout	16.226 (2.632–100.029)	**0.003**

**Table 7:** Diagnostic performance of magnetic resonance imaging in differentiation of idiopathic granulomatious mastitis and breast cancer.

	Sensitivity	Specificity	Accuracy	PPV	NPV
**Model 1 ^1^**	88%	67.65%	78.32%	75%	83.64%
**Model 2 ^2^**	97.33%	55.88%	79.87%	70.87%	95%

^1^ According to the backward stepwise model. ^2^ According to the backward stepwise model + any of the MRI findings (architectural distortion, non-mass enhancement with diffuse distribution or clumped internal enhancement pattern).

## Data Availability

The data are not publicly available due to ethical reasons.
